# Gamification of Dietary Decision-Making in an Elementary-School Cafeteria

**DOI:** 10.1371/journal.pone.0093872

**Published:** 2014-04-09

**Authors:** Brooke A. Jones, Gregory J. Madden, Heidi J. Wengreen, Sheryl S. Aguilar, E. Anne Desjardins

**Affiliations:** 1 Department of Psychology, Utah State University, Logan, Utah, United States of America; 2 Department of Nutrition, Dietetics, and Food Sciences, Utah State University, Logan, Utah, United States of America; 3 Center for Human Nutrition Studies, Utah State University, Logan, Utah, United States of America; 4 Bear River Charter School, Logan, Utah, United States of America; University of St Andrews, United Kingdom

## Abstract

Despite the known health benefits of doing so, most US children do not consume enough fruits and vegetables (FV). School-based interventions can be effective in increasing FV consumption, but the most effective of these require that schools allocate their time, effort, and financial resources to implementing the program: expenditures that schools may be reluctant to provide in climates of academic accountability and economic austerity. The present demonstration project used a behaviorally based gamification approach to develop an intervention designed to increase FV consumption while minimizing material and labor costs to the school. During the intervention, the school (N = 180 students in grades K-8) played a cooperative game in which school-level goals were met by consuming higher-than-normal amounts of either fruit *or* vegetables (alternating-treatments experimental design). School-level consumption was quantified using a weight-based waste measure in the cafeteria. Over a period of 13 school days, fruit consumption increased by 66% and vegetable consumption by 44% above baseline levels. Use of an alternating-treatment time-series design with differential levels of FV consumption on days when fruit *or* vegetable was targeted for improvement supported the role of the intervention in these overall consumption increases. In post-intervention surveys, teachers rated the intervention as practical in the classroom and enjoyed by their students. Parent surveys revealed that children were more willing to try new FV at home and increased their consumption of FV following the intervention. These findings suggest that a behaviorally based gamification approach may prove practically useful in addressing concerns about poor dietary decision-making by children in schools.

## Introduction

Most children in the US do not consume the recommended amounts of fruits and vegetables (FV) on a daily basis [1], [2]. These dietary decisions are a public health concern because FV are rich in vitamins and minerals and have been associated with long-term health benefits such as a reduced risk of hypertension, coronary heart disease, some types of cancer, and stroke [3]. In addition, consuming the recommended amounts of FV may play a role in helping children and adults to maintain an appropriate body weight [4], [5]. FV have low energy density and are often high in fiber and consuming them can produce satiety that may decrease the consumption of calorie-dense, nutrient-poor foods [6].

A wide variety of school-based programs have been implemented with the goal of increasing FV consumption among elementary-school aged children. According to a recent meta-analysis, interventions that provide access to or education about FV tend to not produce the large and lasting increases in FV consumption that are required to impact public health [7]. By contrast, what Evans et al. referred to as “multicomponent interventions” tended to produce larger increases in FV consumption than education- or access-based interventions; however, many of the studies reporting these outcomes rely exclusively on children's self-reported FV consumption [8]–[10]. Because of the children's experience with the intervention (clearly designed to increase their FV consumption), concerns about the Hawthorne effect (sometimes referred to as the “good subject effect;”) [11], [12] influencing self-reports in a direction desired by the experimenter diminishes confidence in the outcomes of these studies. Of those studies that objectively measured FV consumption, the most effective approaches have used a combination of role-modeling and tangible rewards for the repeated tasting of FV [13]–[20]. For example, children participating in the Horne et al. [16], [17] studies watched videos of heroic role models as these characters derived benefits from consuming FV. Concurrently, participating children who consumed FV earned tangible prizes as rewards. This combination of role models and rewards for repeated tasting produced 45–73% increases in FV consumption in these studies.

As noted by Hoffman et al. (2010) [14], two shortcomings of this latter, multicomponent approach are its labor and material costs and lower probability of being implemented properly. Specifically, teachers and cafeteria staff may not have time to carry out tasks such as showing videos, managing a token reinforcement program, or monitoring children's consumption of FV. Hoffman et al. decreased the material costs of their multicomponent approach by using free videos produced by a fruit company and inexpensive stickers as rewards for consuming FV. Objectively measured FV consumption increased in the first year (Cohen's *d* = 0.86 for fruit and 0.34 for vegetables) but by the second year of the intervention vegetable consumption had returned to baseline levels despite the continued implementation of the multicomponent intervention. By the end of the third year when the intervention was no longer in place, both fruit and vegetable consumption returned to baseline levels (Hoffman et al., 2011) [15]. Although material costs of the intervention were low, significant labor costs remained (e.g., each day in the cafeteria, 1 staff member per 30 children was required to observe and reward FV consumption). Nonetheless, implementation fidelity was generally high and school staff rated the program as highly acceptable.

The present study was conducted to further reduce the material and labor costs of a multicomponent intervention designed to increase FV consumption in an elementary school. Like past multicomponent interventions, we used role models [21] and operant reinforcement contingencies [22] to encourage FV consumption. To address concerns about the material costs of tangible rewards (in addition to concerns about the possible negative side-effects of such rewards) [23], a gamification approach was taken in which rewards were virtual – existing only in the game. Gamification describes efforts to use effective video-game design principles to influence workplace and/or socially significant human behavior [24]. A well-designed video game will provide, for example, a compelling narrative in which a character(s) under the player's control completes quests, earns in-game currency, and purchases in-game equipment to aid in these quests. Compelling video games adjust to the skill level of the player so that the game plays as neither too easy nor too difficult. In the gamification intervention employed here, the school played a cooperative game in which, by consuming FV, they helped hero characters to complete quests to find and capture a band of evil villains, to earn virtual currency, and to purchase virtual equipment that aided in their quests. The difficulty level of the game was designed to be neither too easy nor too difficult. To achieve this, virtual rewards were obtained when the school met a daily fruit or vegetable consumption goal, and that goal was set at the 60^th^ percentile of the range of consumption during the preceding 10 days. Thus, the students at the school had consumed the amount of the goal or greater on 4 of the last 10 days.

In addition to reducing material costs by using virtual rewards, our gamification intervention was designed to reduce labor costs relative to other multicomponent interventions. Classroom time spent watching role-model videos was replaced by placing the role models in the brief science-fiction episodes that were read by teachers to their students. Because reading to students is an important part of elementary education, the labor requested of teachers was time spent engaged in a curricular-consistent activity. In the cafeteria, labor costs were reduced by using a school-wide waste-based measure of FV consumption. By having children sort their FV waste into color-coded bins, we quantified daily consumption by comparing FV-supply weights to FV-waste weights. Because we had access to only one school, we employed an alternating-treatments time-series experimental design to evaluate the effects of the game on FV consumption [25].

## Methods

### Ethics Statement

All procedures involving human subjects were approved by the Utah State University Institutional Review Board (USU IRB). An opt-out consent procedure was used in which all students participated unless a parent or legal guardian returned the consent form indicating that (s)he did not want the student to participate. Students who were opted out (*n* = 3) were not included in data collection procedures such that informed consent was obtained from all participating subjects. Written informed consent was not required; our opt-out, passive consent was approved by the USU IRB because of the group curricular aspect of the intervention and the extremely low risks to participants within data collection procedures (see *General Procedures*).

### Participants and Setting

All kindergarten through 8^th^-grade students (N = 180, minus students who were absent on any given day) enrolled at a charter school in Northern Utah were invited to participate in the program. Kindergarteners were 5–6 years old during the study, and each subsequent grade was one calendar year older than this. The charter school did not participate in the US Department of Agriculture's National School Lunch Program (NSLP), though a portion of fruit and vegetables was included with all purchased school lunches each day. The student body was comprised of 54% boys and 46% girls; 87% of students were Caucasian, 6% Hispanic, and 5% Asian.

### Materials

A 317-kg capacity scale with a resolution of 100 gm was used to measure food weights (LW Measurements, LLC; Santa Rosa, California). A smaller scale with a resolution of 1 gm was used to measure portion weights (Ozeri; San Diego, California). Different-colored 62.5-liter storage bins were used as fruit- and vegetable-waste receptacles. A game display measuring 2.1×1.1 m, made from colored poster boards, was mounted approximately 1.5 m above the floor on a wall in the cafeteria; icons for the game display were created using commonly available materials (e.g., construction paper, pipe cleaners). Poster boards (55.9×71 cm) were used on days when participating children voted on the direction of the game narrative.

### Procedure

#### General procedures

Throughout the study, one variety of fruit and one variety of vegetable (see Table 1 for varieties) were served daily to all students according to the pre-planned school lunch menu. A total of five varieties of fruit (three fresh and two canned) and five varieties of vegetables (three fresh and two canned) were served throughout the study. Students who brought lunch from home were allowed to take servings of the FV at no cost; parents and students were informed of this prior to the onset of the study. Fruits and vegetables were provided in volumetric servings (just under ¼ cup for K-2 grade, approx. ¼ cup for 3–5 grades, and approx. 1/3 cup for 6–8 grades). Students were allowed to return to the serving area to take additional servings of fruits, vegetables, or both. Upon finishing lunch, students placed their FV waste into the differently colored fruit- and vegetable-waste receptacles; one cafeteria staff member supervised students in this activity throughout the experiment.

**Table 1:** Daily Consumption Goals and the Fruit and Vegetable Served each Day in the Gamification Phase.

Day	Consumption Goal (cups)	Consumption Goal (gm)	Target Food(s)	Non-target Food	Goal Met?
1	0.15	27	Mandarin Oranges	Salad	Yes
2	0.15	24	Oranges	Veggie Sticks	Yes
3	0.11	12	Veggie Sticks	Bananas	Yes
4	0.15	39	Applesauce	Carrots	No
5	0.15	23	Bananas	Carrots	Yes
6	0.11	12	Veggie Sticks	Oranges	Yes
7	0.11	13	Carrots	Apples	Yes
8	0.15	25	Oranges	Corn	No
9	0.15	33	Peaches	Salad	Yes
10	0.15	29	Mandarin Oranges	Salad	Yes
11	0.12	13	Veggie Sticks	Oranges	Yes
12	0.15	39	Applesauce	Carrots	Yes
13	0.12	18	Green Beans	Oranges	Yes

Note: Veggie sticks were raw carrots and celery.

Daily school-wide consumption of fruit and vegetable were calculated separately using a weight-based measure: 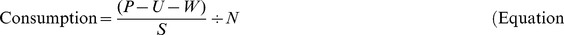
(Equation \;1)where *P* is the total weight of the supply of fruit or vegetable prepared for serving that day, *U* is the weight of the unserved supply of fruit or vegetables, *W* is the weight of the fruit or vegetable waste collected in the lunchroom waste receptacles, *S* is the weight of a single serving of fruit or vegetable, and *N* is the number of children in attendance on the school day. The numerator of Equation 1 yields school-wide consumption, the denominator converts this to the number of single servings consumed, and dividing by the number of students in attendance provides a between-student average proportion of a serving consumed. On days when salad or vegetable sticks were served, the weight of the salad dressing used was subtracted from the numerator of the vegetable consumption equation (a subtraction that erred on the side of *underestimating* the amount of vegetables consumed because all numerator subtractions translate to less consumption). Inedible portions of fruit (orange peel and banana peel) were removed prior to weighing the single servings (i.e., *S*).

#### Baseline

Baseline fruit and vegetable consumption were measured across a ten-day baseline using the procedures outlined above. Consumption of fruit and vegetables decreased over the baseline revealing either a reactivity to measurement effect [26], [27] or a reduction in novelty to the availability of unlimited free fruits and vegetables to all students. Because consumption of vegetables was stable over the final five days of baseline (runs test indicated the slope of the regression line did not significantly deviate from zero, *p* = .84), vegetable consumption during the Gamification phase was compared to these final five days of baseline. Fruit consumption continued to decline over the final five days of baseline. Because the intervention was anticipated to reverse this trend, the Gamification phase was initiated despite this continued decline in fruit consumption. The final five days of baseline fruit consumption was used for comparison purposes with data from the Gamification phase.

#### Gamification phase

An alternating-treatments experimental design was employed throughout the Gamification phase. Each day, the intervention sought to increase either fruit consumption *or* vegetable consumption by giving the school a goal to consume more of the food targeted for an increase on that day. The targeted food (fruit or vegetable) was randomly selected with the constraint that no food category could be selected on more than three consecutive days.

Goals were set daily using a percentile schedule of reinforcement [28] with the goal being the 60^th^ percentile of the preceding 10 days' consumption. For example, if the targeted food was fruit, the prior 10 days of fruit consumption on fruit-target days were rank-ordered and the 60^th^ percentile of this array of values served as the goal. On each new day in which fruit, for example, served as the target food, a new fruit goal was calculated using the same procedure except that the oldest fruit-consumption data point in the array was discarded and replaced with the amount of fruit consumed on the last day that fruit was the targeted food. This was designed to gradually increase the consumption goals over the course of the phase. Table 1 shows the targeted foods and quantitative consumption goals on the 13 days of the Gamification Phase.

Daily consumption goals were communicated to students by instructing them to eat more fruit *or* vegetable than they would normally consume during lunch (no specific amounts [e.g., half a serving] were mentioned because this goal might be too difficult for some [e.g., vegetable refusers] and might lower consumption in others [those who already consume full portions of FV]). The first of these goals was communicated during a school-wide assembly held just before lunch on the first day of the Gamification phase. During the assembly, the heroic and villainous characters were introduced and students were told that over the next few weeks they would play a game in which they could help the heroic characters to capture each of the villains. This help would come in the form of energy that the students could harness for the heroes by eating fruits or vegetables in the cafeteria. On subsequent days, just before lunchtime, teachers identified the target food (fruit or vegetable) and encouraged them to eat more of it than normal.

At the conclusion of the assembly that opened the Gamification phase, students had the opportunity to visit seven tasting stations where small portions of three fruits and four vegetables were served. Five of the seven fruits and vegetables were those that regularly appeared on the school lunch menu. Students who consumed six of seven tasting portions earned a small prize (e.g., temporary tattoo), and students who consumed all seven foods earned a small prize plus a large prize (e.g., mechanical pencil, flying disc, etc.). Tangible rewards were arranged at the beginning of the intervention because we anticipated that virtual rewards might be insufficient to encourage some children to try foods that they normally avoided. No other tangible rewards were used for the remainder of the study.

Throughout the remainder of the Gamification phase, when goals were met, on the next school day and just before lunch classroom teachers read to their students the next episode (approximately 3 min in duration) of a science-fiction adventure story that was written for the purpose of this study by the second author of this paper (available upon request; greg.madden@usu.edu). Each episode described the exploits of the heroic characters as they attempted to find and capture the villains. Each episode concluded by encouraging students to eat more of the targeted food than normal so that the heroes would have enough energy to continue their struggles against evil. If the school failed to meet a goal, no new episode was read; instead, teachers read a message from the fictional heroes that encouraged them to eat more of that food than normal. When the first consumption goal was met, the first villain was captured on a planet chosen by the school. The second villain was captured after the 8^th^ goal was met and the game concluded after meeting the 11^th^ goal, and with the capture of the third and most fearsome of the villains (i.e., the boss battle).

A game display made of construction paper and commonly available art materials was posted on the wall of the cafeteria. The display showed hand-drawn depictions of the planets to which the heroes travelled within the narrative, the villains captured during the course of the game, and the cumulative amount of a game currency that the school had earned. Game currency was earned by exceeding the daily quantitative consumption goal; one currency unit was awarded for every 1% of a portion by which the goal was exceeded. A research assistant updated the game display daily before lunch.

On Days 2-4, 6, and 11-13 of this phase, students voted in the cafeteria to influence events happening in the narrative episodes. For example, students sometimes voted on the planet on which to search for the villains. The planet that received the most votes was inserted into a blank placeholder space in the pre-written episode to be read the next day (e.g., “Wow, you met your goal to eat more vegetables than normal so the heroes flew their ship to the ______ planet”). This allowed use of the same episode regardless of the outcome of the vote. As a second example, on the final day of the Gamification phase, students voted on which tool to purchase with their accumulated game currency (e.g., a tornado gun, a dirty-sock cannon). During voting, each student made a check mark on a poster board near hand-drawn or clip-art depictions of the alternatives (e.g., the three nearby planets). A research assistant supervised voting.

#### Satisfaction Surveys

At the end of the Gamification Phase, teachers, parents, and students were encouraged via e-mail notification to complete an online satisfaction survey. The student response rate (<5%) was too low to interpret. The other surveys of teachers and parents are shown in Table 2. Both surveys used a five-point Likert scale where 1 =  Strongly Disagree, 3 =  Neither Agree nor Disagree, and 5 =  Strongly Agree. The exception was the final two questions of the parent survey, which asked parents to categorize their child's daily FV consumption before and after the intervention (1 =  less than one cup, 2 = 1–1.5 cups, 3 = 2–2.5 cups, 4 = 3–3.5 cups, 5 = 4 or more cups).

**Table 2:** Teacher (n = 7) and Parent (n = 35) Satisfaction Surveys.

Teacher Survey			Median	Low Score
I read the episodes to my class every day			5.0*	2
Students enjoyed the episodes			5.0*	2
Student behavior/concentration has improved			3.0	3
My FV consumption has increased			4.0	2
The program would be beneficial to other schools			4.0*	4
Parent Survey				
My child enjoyed the episodes			4.0**	2
My child enjoyed the change in school culture toward eating FV			4.0**	3
My child consumed more FV at school			4.0**	3
My child consumed more FV at home			4.0**	2
My child was more willing to try new FVs			4.0**	3
My child asked me to buy more of a specific FV			3.0	1
My child's behavior/concentration has improved			3.0	1
My child's general health has improved			3.0	1
My FV consumption has increased			3.0	1
I am happy with results and believe other schools would benefit			5.0**	3

*Lower 95% CI of median >3 (Neither Agree nor Disagree).

**Significantly different from 3; p<.0001, Wilcoxon Signed-Rank Test.

#### Data Analysis

All statistical analyses were performed using the proportion of portions metric yielded by Equation 1. These values were also converted to grams (weight-based measure) and cups (volume-based measure) after the study was completed for reporting purposes. Some varieties of fruits and vegetables weighed different amounts despite similar volumes; for example, a ¼-cup serving of fresh salad weighs 14.25 grams, and a ¼-cup serving of canned green beans weighs 38.25 grams. Examining changes in consumption as measured by weight (grams) may be misleading because of these weight differences. For example, large increases in salad consumption volume would translate to small increases in vegetable consumption weight compared to small increases in green bean consumption volume.

To evaluate the effects of the game-based intervention, we first used a Simulation Modeling Analysis (SMA) to determine if post-baseline fruit and vegetable consumption (analyzed separately) increased above baseline levels in the Gamification phase. The two time-series of fruit and vegetable consumption included all post-baseline days (i.e., vegetable consumption on days in which fruit was targeted by the game were included in the vegetable time series). This analysis evaluated if FV consumption increased significantly despite only one food being targeted for change on most days of the Gamification phase.

The SMA is appropriate for brief time-series data because it takes into consideration autocorrelation within the data stream [29]. An ANOVA is inappropriate for time-series data. Likewise a generalized estimating equation could not be used because i) the baseline was too brief and ii) only one school participated in the study. Briefly, the SMA estimates autocorrelation in the obtained baseline and intervention phases and corrects for small-n bias [30]. It then obtains a Pearson correlation coefficient (*R*) between the obtained time-series data and the dummy-coded (0 and 1) baseline and intervention phases. Spearman rank-order correlation coefficients may also be used within the SMA. We report Pearson's coefficient values because the statistical significance of the outcome was unaffected by the correlation coefficient selected. The SMA then randomly generates 5000 random-normal time-series data streams with the same autocorrelation and the same number of observations in each phase as the observed data. The proportion of randomly generated data streams with a correlation coefficient (against the phase vector) greater than or equal to the obtained correlation coefficient serves as the *p*-value.

The second analysis was designed to evaluate if the Gamification intervention was responsible for increased consumption of FV. If it was, then on days when fruit (vegetable) consumption was targeted by the game, fruit (vegetable) consumption should be significantly higher than during baseline *and* vegetable (fruit) consumption should not be significantly elevated relative to baseline. To evaluate the role of the intervention on consumption we used the Conservative Dual Criteria (CDC) method developed by Fisher, Kelly, and Lomas [31]: a method developed using Monte Carlo simulations to yield acceptable power and low rates of Type I error with time-series data sets as small as five observations in baseline and treatment. The two criteria in the CDC are binomial tests that determine if the treatment data are significantly elevated above (i) the baseline mean plus 0.5 standard deviations (i.e., a moderate effect size) and (ii) the baseline-trend-predicted level (assessed via linear regression) elevated by 0.5 standard deviations. Applied to our data, if binomial tests indicated that consumption of the target food was significantly higher than the baseline level (+0.5 SD) and the projected trend (+0.5 SD), then both criteria in the CDC were satisfied and the difference was considered significant. We predicted that the consumption of FV would meet the dual criteria only on days when that particular food (fruit or vegetable) was the target food.

For parent post-intervention satisfaction surveys, a Wilcoxon's Signed-Ranks test was used to determine if item ratings deviated significantly from 3 (the response indicating neither a positive nor negative opinion). Although the proportion of teachers that completed their satisfaction survey was high (87.5%) the number of teachers in the sample was small (N = 8). Therefore, if a single teacher provided a rating at or below 3 the Wilcoxon's test was rendered nonsignificant. To avoid this overly conservative criterion, item ratings were considered significantly higher than 3 if the lower 95% confidence interval (CI) was greater than 3.

## Results

**Figure 1:**
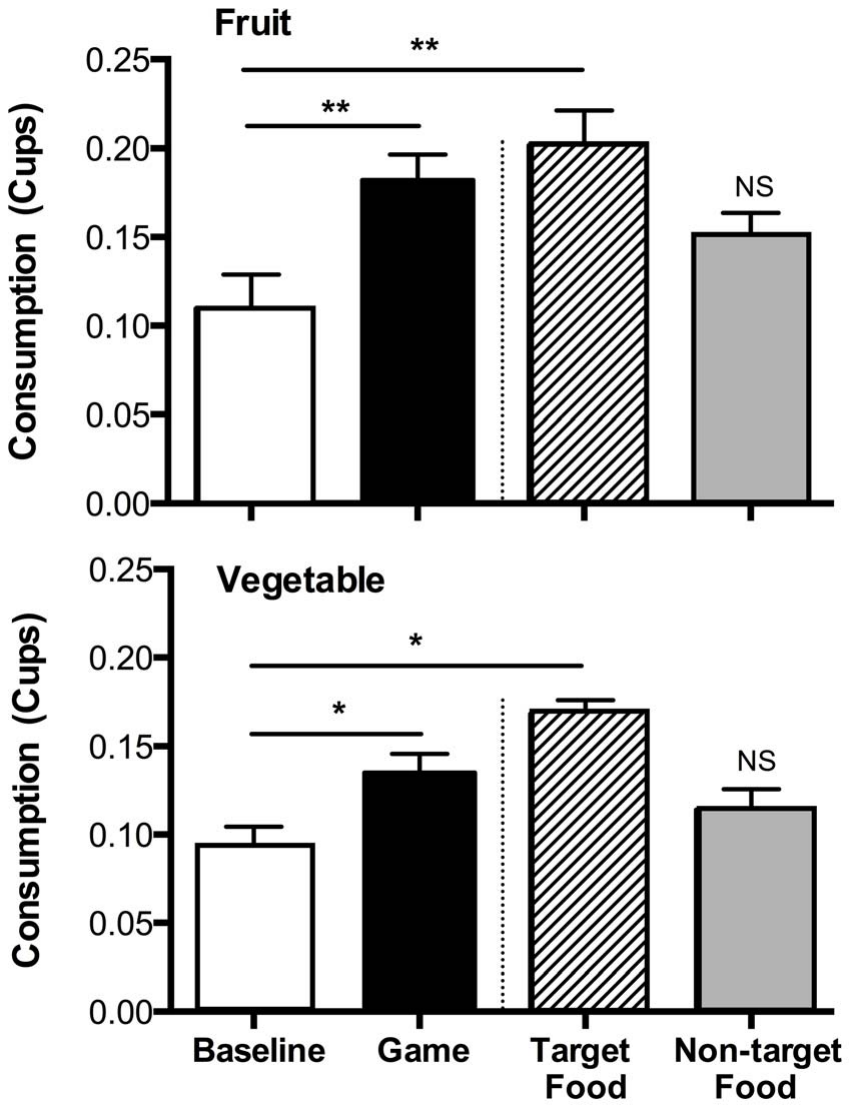
Fruit and Vegetable Consumption Across Baseline and Gamification Phases. The left sides of the panels show fruit (top panel A) and vegetable (bottom panel B) consumption (in cups) from the last 5 baseline days and all 13 days of the Gamification Phase. The right side of each panel separates consumption on days when the food indicated in the panel was targeted by the intervention for increased consumption (hatched bar) and on days when that food was not targeted (grey fill bar). * *p*<.05. ** *p*<.01.

Figure 1 shows the average (+ SEM) cups of fruit and vegetable consumed per day in the Baseline and Gamification phases (all data may be obtained from the second author upon request). The right side of each panel separates consumption on those days when the target food was the food indicated in each panel. Baseline levels of fruit and vegetable consumption were 0.11 cups (17.71 gm) and 0.09 cups (11.41 gm), respectively. The SMA indicated that fruit (*R* = .57, *p*<.01) and vegetable (*R* = .48, *p*<.05) consumption increased significantly following the Baseline phase. The *R* values are Pearson's correlation coefficients obtained when consumption data are plotted as a function of the dummy coded baseline (0) and intervention (1) phases. Recall that this analysis ignored what food (fruit or vegetable) was targeted by the intervention, evaluating instead the overall level change following the Baseline phase (i.e., the left sides of the graphs in Figure 1). During the Gamification phase, fruit consumption increased by 66% to an average of 0.18 cups (32.56 gm, an 84% increase) per day. Similarly, vegetable consumption increased by 44% to an average of 0.13 cups (14.65 gm, a 28% increase) per day.

The CDC analysis revealed that consumption of fruit (*p*<.01) and vegetables (*p*<.05) were significantly higher than baseline on days when these were the target foods during the Gamification phase. Consistent with the hypothesis that the contingent relation between target-food consumption and game-based rewards was responsible for elevated consumption, there was no significant increase in fruit or vegetable consumption when these foods were the non-target foods (i.e., the foods unassociated with goals and game-based rewards; both *p*'s>.5).

The results of the post-intervention teacher and parent satisfaction surveys are presented in Table 2. Response rate was high for teachers (87.5%) and moderate among parents (23%, a response rate that falls well within the range of rates empirically demonstrated to produce valid outcomes when compared with higher response rates) [32]. Noting only the significant findings, most teachers indicated that they were able to read the episodes in their class, the students enjoyed the episodes, and they believed the program would be beneficial if implemented at other schools. The principal of the school invited our research team to return to the school next year to play the FV game again. Among parents, several survey items obtained scores significantly greater than 3 (e.g., my child enjoyed the change in school culture toward eating FV). Of greatest interest was that parents reported that after the intervention their children were consuming more FV at home and that they were more likely to try a new FV. Parents were highly satisfied with the intervention and indicated it would be beneficial if implemented at other schools. On those survey questions that asked parents to estimate their child's FV consumption before and after the gamification intervention (not shown in Table 2), parents indicated that consumption had increased by an average of 0.41 cups per day and a Wilcoxon Signed Rank test applied to pre-post difference scores indicated that this increase was significantly different from zero (*p*<.01).

## Discussion

Cafeteria-based FV consumption among K-8^th^ grade students increased significantly above baseline levels when a low-cost, behaviorally based gamification intervention was introduced. Across all days of the intervention, fruit and vegetable consumption increased above baseline levels by 66% and 44%, respectively when measured in cups, and 84% and 28%, respectively when measured in grams. Importantly, because consumption of the targeted food (fruit or vegetable) increased significantly above baseline levels but consumption of the non-targeted food did not, the overall increases in FV consumption may be attributed to the efficacy of the intervention. After the intervention had ended, teachers indicated that other schools would benefit by playing the game. Likewise, parent responses were significantly positive on survey items inquiring about FV consumption and satisfaction with the school-based intervention. Therefore, the goal of positively impacting children's dietary choices at school, and to do so in a fun, low-cost, low-labor fashion, was achieved.

Although FV consumption was increased by the gamification intervention, the amounts of FV consumed still fell below the per-meal amounts recommended by the USDA. Where the USDA recommends children grades K-8 consume 0.5 and 0.75 cups of fruit and vegetables, respectively at lunch, our participants consumed an average of 0.18 and 0.13 cups, respectively during the gamification phase. Even when nontarget days are excluded (because the game targeted the other food for increased consumption), fruit (0.2 cups+0.05) and vegetable (0.17 cups+0.02) consumption were still below the USDA guidelines.

The gap between baseline levels of FV consumption and the USDA guidelines might be further decreased if the game were played for a longer duration. Daily consumption goals were set modestly above recent consumption (always the 60^th^ percentile of the last 10 days' consumption) and were updated daily. Assuming that the school would continue to meet its consumption goals during a longer version of the game, this dynamic goal-setting algorithm would continue to gradually increase the consumption goal until consumption approximates the USDA standards. An empirical research base supports the use of these percentile schedules of reinforcement [28] for producing gradual changes in socially significant behavior [33]–[35]; the technique is used in basic behavioral pharmacology and toxicology research with nonhuman animals [36]–[38]. Investigating the efficacy of a longer version of the gamification intervention should be a direction for future research, while maintaining the goal of minimizing its material and labor costs.

### Minimizing Material and Labor Costs

As noted earlier, multicomponent school-based interventions are effective in improving healthy eating in schools [8]. However, these multicomponent interventions may require the purchase of materials (e.g., stickers, videos, tangible rewards) and always require some amount of teacher labor (e.g., passing out stickers during lunch [14], [15]; managing a point system for delivering intermittent tangible rewards[16], [17], [19]). In our gamification intervention material costs were minimized. The game display that hung in the cafeteria (and perhaps served to remind children of the game and their goal to consume more FV) was made of construction paper and other readily available art supplies. Although our research team made the game display and updated it daily, children in an art class and/or in a before/after-school club could undertake these activities. Because all of the rewards were delivered within the narrative (e.g., learning if a villain was captured by searching on a planet) or on the game display (e.g., game currency) the material costs of the game-based rewards were nominal.

Teacher labor in the current study was confined to reading the science-fiction episodes before lunch (approximately 3 min for 13 days) and, as noted above, teachers reported that they were able to complete this task daily. Nonetheless, future game-based interventions should seek to lower this labor cost, as it may be a barrier to school adoption. One solution is to play audio-recorded versions of the episodes over a school-wide public-address system. This would allow teachers to pursue other academic-preparatory tasks while potentially increasing the production quality of the episodes. Perhaps the largest labor-cost of the present intervention was placed on the kitchen staff, who was asked to weigh FV before and after lunch. In addition, if a school were to implement this intervention without outside assistance, they must allocate one cafeteria employee to monitor children's sorting of FV waste. Although weighing tasks were typically completed in less than 10 minutes, and monitoring the sorting of FV waste is not onerous, some schools may not agree to these reallocations of labor. A low-labor alternative would be to install automated tray-photo stations where pictures of pre- and post-lunch trays are taken and advanced software estimates FV consumption [39]. Of course, this low-labor approach increases the materials cost of the intervention.

### Other Limitations

Four other limitations of this pilot project are noteworthy. First, the intervention was conducted in a single charter school in Utah that did not explicitly follow the USDA's NSLP guidelines and were thus serving smaller amounts of FV than typical schools. Whether the intervention would work as well in other larger, culturally more diverse, or rural schools is unknown. Likewise, the acceptability of the program to other teachers and parents cannot be evaluated from this single-school study. Answering these questions of generality and between-school replicability will have to await future studies.

Second, the intervention began with a school-wide tasting session in which children earned tangible rewards for consuming small portions of FV. Some children may have anticipated that additional tangible rewards would be obtained for consuming FV during the gamification phase, and this may have played a role in the significant increases in FV consumption. Three pieces of evidence argue against this. First, the narrative episodes made it clear that game based rewards (e.g., new episodes, game currency) were the only rewards for increased FV consumption. Second, if tangible rewards were anticipated but never delivered, one would expect a decreasing trend on FV consumption during the intervention phase. No such decrease was detected by runs tests applied to the slope of regression lines fit to the time-series fruit (*p* = .36) or vegetable (*p* = .93) data from the gamification phase. Third, in a recently completed systematic replication of our game-based intervention (unpublished data), we obtained significant increases in FV consumption when no tangible rewards were used at any time during the gamification phase.

A third limitation is that the school-wide consumption measure developed for this study did not allow us to evaluate the effects of the intervention on the FV consumption of individuals. Thus, the source of the increased consumption is impossible to identify: did all students consume more than normal or did a smaller group of students drastically increasing their consumption? When rewards are given to the group based on the collective performance of the group, there may be a tendency for some within the group to exert less effort; i.e., social loafing [40]. At present, we know only that FV consumption increased significantly following the intervention and that the intervention was responsible for this increase; we do not know how this increased consumption was distributed across individual children. As above, automated analysis of lunch tray photos offer one avenue for addressing this limitation.

Fourth, the duration of the intervention was brief, so we do not know if the increased levels of FV consumption could be maintained if the duration of the Gamification phase were extended by, for example, playing a game that required more accomplishments be made before the ultimate goal was met. For example, a number of industry-based gamification interventions involve earning virtual trophies and/or leaderboards. One way in which the present game could be extended would be to place the school on a leaderboard with fictional schools from, for example, other planets, and with whom the school competes for trophies and to qualify to play the science fiction game played in the present study.

### Learning Theory and Gamification

The gamification intervention employed here is theoretically grounded in social learning theory [21] and operant learning theory [22]. Role-model heroes encouraged students to consume more FV and when students met these goals, a variety of game-based reinforcers were delivered. The game context adds to the incentive-based approach by providing a platform for delivering low-cost virtual reinforcers for FV consumption. That platform was the science-fiction adventure game and the reinforcers were the episodes that teachers read to their students, the capturing of villains, the acquisition of virtual currency and the goods purchased with that currency, etc.

Gamification proponents [24] have argued that the game-design techniques used by video-game programmers can improve the efficacy of behavior-change interventions. A potentially important one of these techniques is to create a compelling narrative in which the game is played. In our game, the narrative pitted the heroes against the villains and enlisted the school in this battle. The narrative clearly established the object of the game (find and capture the villains) and clearly connected player behavior to game outcomes (if the school meets its FV consumption goal, then episodes in the narrative will be read, currency will be earned, villains will be captured, etc.). Within behavior analysis, these functions of the narrative are identified as *establishing operations* [41]; that is, stimulus changes that enhance the value of a consequence as a reinforcer. The science fiction adventure episodes were often written with cliff-hanger endings designed to enhance the value of a virtual reward. For example, one episode ended with a giant eating the hero's spaceship, leaving them stranded on a planet with no communication abilities except for that with the school. However, if the school met their consumption goal on that day, the heroes could purchase a new ship with the virtual currency earned. An empirical challenge for future research is to quantify the value-enhancing effects (if any) of contextualizing reinforcers within a game narrative. A trinket reward, like a rubber ball, may have reinforcing value, but can the value of that reward be enhanced if the student must earn the rubber ball before a hero in the game-narrative may acquire the same ball and use it in a battle with a high-level villain? The widespread sale of toys that appear in cartoons and video games and the purchasing with real money of virtual items earned within video games [42] suggests that virtual rewards have quantifiable value that may enhance or replace more costly incentive-based interventions.

As previously mentioned, another question for future research is if using game-based virtual rewards can enhance student interest in the game and, as a result, can sustain increases in FV consumption in longer duration interventions. Video game programmers use the acquisition of virtual rewards to increase the probability of sustained game play. For example, when a player earns a magic scroll or unlocks a new area of the game, the player may be less likely to quit because they want to use the scroll or to explore the new area of the game. Translated to a gamified intervention, virtual rewards may enhance engagement with the game, with the characters in the game, and with the goal of consuming more FV. How long increased FV consumption can be maintained is an empirical question. Designing an effective game will employ principles of social learning theory (e.g., role models), behavior analysis (e.g., schedules of incentives, establishing operations, token economies, etc.), and newly emerging principles of gamification [24].

## Conclusions

Because a) the majority of children in the US do not consume recommended amounts of FV [1], b) the health benefits of doing so are well established [3], c) some evidence suggests eating FV plays a role in maintaining an appropriate body weight [4], and d) schools offer a venue in which more than 30 million US children consume at least one important meal each day; developing and empirically evaluating practical, low-cost, low-effort, school-based interventions should be a national priority. The present study demonstrates the initial feasibility and efficacy of a gamification-based intervention for increasing school-wide FV consumption.
